# Gene Co-Expression Network Analysis for Identifying Modules and Functionally Enriched Pathways in Type 1 Diabetes

**DOI:** 10.1371/journal.pone.0156006

**Published:** 2016-06-03

**Authors:** Ignacio Riquelme Medina, Zelmina Lubovac-Pilav

**Affiliations:** Bioinformatics research group, School of Biosciences, University of Skövde, Skövde, Sweden; University of São Paulo, BRAZIL

## Abstract

Type 1 diabetes (T1D) is a complex disease, caused by the autoimmune destruction of the insulin producing pancreatic beta cells, resulting in the body’s inability to produce insulin. While great efforts have been put into understanding the genetic and environmental factors that contribute to the etiology of the disease, the exact molecular mechanisms are still largely unknown. T1D is a heterogeneous disease, and previous research in this field is mainly focused on the analysis of single genes, or using traditional gene expression profiling, which generally does not reveal the functional context of a gene associated with a complex disorder. However, network-based analysis does take into account the interactions between the diabetes specific genes or proteins and contributes to new knowledge about disease modules, which in turn can be used for identification of potential new biomarkers for T1D. In this study, we analyzed public microarray data of T1D patients and healthy controls by applying a systems biology approach that combines network-based Weighted Gene Co-Expression Network Analysis (WGCNA) with functional enrichment analysis. Novel co-expression gene network modules associated with T1D were elucidated, which in turn provided a basis for the identification of potential pathways and biomarker genes that may be involved in development of T1D.

## Introduction

Type 1 diabetes (T1D) is a complex trait, which develops when the insulin producing beta cells are destroyed resulting in a decreased production and secretion of insulin. According to National Center for Chronic Disease Prevention and Health Promotion (CDC), diabetes is one of the most common chronic diseases worldwide, having a prevalence of 9.3% of the population in United States [1]. Furthermore, diabetes is also a major contributor of renal diseases, amputation, cardiovascular disease [2,3], and has been projected to be the seventh leading cause of deaths in 2030 [4].

T1D is a heterogeneous disease with both underlying genetic and environmental factors that influence the development and progression of the disease [5]. Important chromosomal regions that have been shown to contribute to disease susceptibility are the human leukocyte antigen (HLA) region at chromosome 6 and insulin gene region at chromosome 11 [3]. Nevertheless, only small percentage of genetically susceptible individuals progress to clinical disease, which indicates the involvement of environmental triggers.

Previous research in this field has been primarily focused on analysis of single susceptibility genes [6–10] or performing Genome Wide Association Studies (GWAS) to identify genetic determinants of disease [11–15]. In addition, majority of the studies are focused on the beta cells whereas novel findings are pointing to the importance of the immune system in the disease development [16,17]. This study is based on the public data derived from the samples from peripheral blood mononuclear cells (PBMC), involved in innate immune activation that may play both pathological and protective role in T1D [18]. PBMC are suitable for the assessment of immunological markers in T1D children, as stated in earlier study [19].

Current studies on T1D do not take in account the interactions between the genes or proteins, which are crucial for understanding molecular mechanisms underlying complex disease. Recently, importance of network-based approaches to human disease has been highlighted [20]. Cellular interconnectedness effects the disease progression and should be taken into account in the identification of disease genes and pathways, which in turn, may provide new targets for drug development.

In this study, we hypothesize that pathogenesis of T1D is reflected by the perturbation of intercellular and intracellular networks, which may lead to identification of specific disease modules caused by a variation in one or more of the components. We adopted a well-established network-based approach, Weighted Gene Co-Expression Network Analysis (WGCNA) [21] to identify modules in co-expression gene networks that may be associated with T1D. To the best of our knowledge, this approach has not been applied in previous T1D studies. This method, in combination with functional enrichment and network topology measures, is also applied here to identify potential biomarkers for T1D that will aid in the understanding of the mechanisms of T1D.

We identified co-expression modules that show significant disruption, by comparing global co-expression network in T1D to the corresponding network derived from healthy controls. Within the identified co-expression disease modules that were chosen for further analysis, we found several significantly enriched Kyoto Encyclopedia of Genes and Genomes (KEGG) pathways with association to T1D, such as Type I diabetes mellitus, mTOR signaling pathway etc. Besides confirming some genes with previously known T1D involvement, such as fas cell surface death receptor (FAS), interleukin 1 beta (IL1B) and interleukin 8 (IL8), we also identified interesting candidate genes that could be further analyzed further to understand their roles in T1D.

## Materials and Methods

### Affymetrix Microarray

Microarray data GSE9006 from NCBI Gene Expression Omnibus (GEO) database was collected from peripheral blood mononuclear cell (PBMC) samples from 43 children with newly diagnosed T1D and 24 healthy controls [22]. For 20 patients, one and four month follow-up samples were also included in the analysis. The data was normalized using the global scaling normalization method and filter was applied based on Affymetrix flag calls, according to the same procedure as in [22]. Probe sets were selected for further analysis if present in at least 50% of samples in any group using R Bioconductor *affy* package [23]. After the initial filtering, 17,310 genes were left. The data set was preprocessed further by applying Significance Analysis of Microarrays (SAM) [24] to remove genes that show no or very low changes in expression between healthy and disease samples, but also for the purpose of getting manageable size of the data for WGCNA analysis. R Bioconductor package *siggenes* [25] that implements SAM was used (delta = 0.296), leaving the total of 10005 genes in the final set for further analysis.

### Weighted Gene Co-Expression Analysis (WGCNA)

WGCNA is a method for deriving co-expression networks [21], and it is implemented in R *WGCNA* package [26]. The method for constructing network is as follows: first, a similarity co-expression matrix was calculated with Pearson's correlation *cor*(*i*,*j*) for all genes, and transformed to an adjacency matrix (AM) by using the soft thresholding power beta, to which co-expression similarity is raised (se Eq 1).

$$a_{ij}={\left(0.5*\left(1+cor\left(i,j\right)\right)\right)}^{\beta }$$ (1)

where *a*_*ij*_ represents the resulting adjacency that measures the connection strengths.

We chose the power beta based on criteria of approximating scale-free topology of the network, as prescribed in the original publication [26]. Power of beta = 9 was chosen based on the scale-free topology criterion. This criterion shows that the power parameter, beta, is the lowest integer such that the resulting network satisfies approximate scale-free topology (linear regression model fitting index *R*^2^ that should be larger than 0.8).

Then, topological overlap matrix (TOM) [27] was computed from AM, and TOM was in turn converted into a dissimilarity TOM. Finally, hierarchical clustering was used to produce tree (dendrogram) from dissimilarity TOM. By using dynamic tree cutting, different number of clusters (modules) was obtained from the tree. The resulting modules contained genes that are densely interconnected and we constructed two different networks, one using the healthy samples and the other using the T1D samples.

WGCNA can be used for summarizing obtained modules by using concept of eigengene, and further screening for suitable gene targets by calculating module membership (*kME*) measures, also known as eigengene-based connectivity [21,26]. Eigengenes are defined as the first principal component of the expression matrix for each module, and represent the weighted average of the expression profile for each module. The eigengenes can be used to merge clusters with a similar expression profile, leading to the final set of modules as a result of constructing the network.

### Preservation of modules

Module preservation statistics in WGCNA was used to evaluate whether a given module defined in reference data set (healthy network) can also be found in the test data set (disease network). The overall significance of the preservation statistics was assessed using *Z*_*summary*_ Eq (2) that combines multiple preservation statistics into a single overall measure of preservation, which considers both aspects of density and connectivity preservation [28].

$$Z_{summary}=\;\frac{Z_{density}+Z_{connectivity}}{2}$$ (2)

Based on the thresholds proposed in original method proposal [28], resulting *Z*_*summary*_ < 2 indicates no preservation, 2< *Z*_*summary*_ <10 indicates weak to moderate evidence of preservation, and *Z*_*summary*_ >10 means strong evidence of module preservation.

### Pathway enrichment of the significant modules

We performed pathway enrichment analysis of selected modules by using two different tools, a network-based gene set enrichment analysis, EnrichNet (http://www.enrichnet.org/) [29] and meta-database ConcensusPathDB (http://cpdb.molgen.mpg.de/) [30]. This includes enrichment in predefined pathways by, for example,—KEGG (http://www.genome.jp/) and Gene Ontology—GO terms (http://www.geneontology.org/).

### Topological analysis with betweenness centrality measure

Modules obtained from WGCNA can be exported and analyzed with other tools. We performed topological centrality analysis by using R package Igraph [31]. More specifically, betweenness centrality (*BC*) of a node was used as a network topology measure [32]. *BC* is defined as the number of shortest paths between every two other nodes in the module that pass through that node Eq (3).

$$\;BC\left(v\right)=\Sigma_{s\neq v\neq t}\frac{\sigma_{st}\left(v\right)}{\sigma_{st}}$$ (3)

Where *V* is the set of nodes, *σ*_*st*_ is the number of shortest paths between nodes *s* and *t*, and *σ*_*st*_(*v*) is the number of those paths that pass through node *v*.

Simply stated, it measures the relevance of a node (gene) as capable of transferring communication between the genes in the module. High values of *BC* indicate “high traffic nodes”, and hereby more biologically informative nodes in a module.

### Visualization and exploring modules with VisANT

Module visualization and further analysis was performed with VisANT (http://visant.bu.edu/) software, which allows visualization and analysis of networks of biological associations and interactions [33].

## Results

### Co-expression network generation with WGCNA

**Fig 1:**
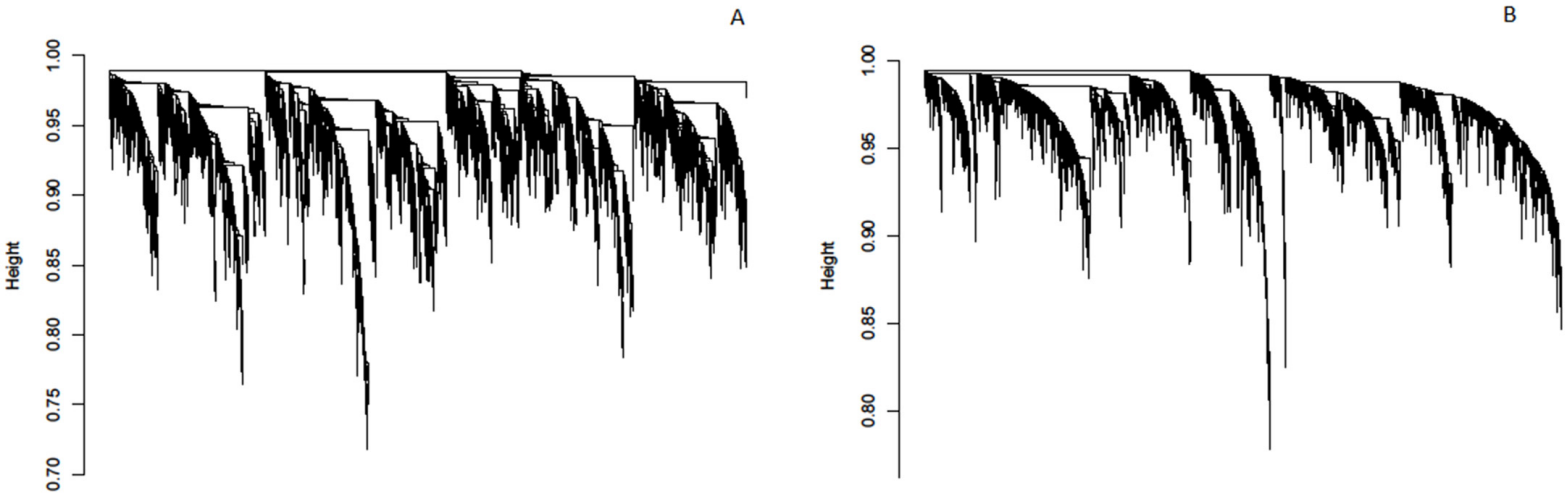
Gene dendrogram generated with WGCNA for (A) healthy samples and (B) T1D samples. Each leaf (vertical lines) in the dendrogram corresponds to a gene.

The details of the gene co-expression network construction with WGCNA are explained in [21]. By applying the steps described in Materials and Methods, two different networks were generated; one for 24 healthy samples and the other for T1D samples (43 samples, and for 20 patients there are replicates—1 month and 4 months after diagnosis). Briefly, signed network adjacency matrices were obtained by raising the Pearson correlation matrices to a power beta = 9 which approximates scale-free topology. The adjacencies were transformed to dissimilarity matrix for subsequent average linkage hierarchical clustering using *flashClust* R package [34]. Resulting trees (dendrograms) are illustrated in Fig 1A (healthy samples) and Fig 1B (T1D samples) and each leaf (vertical lines) corresponds to a specific gene. This illustration is intended to show apparent changes in tree structures between two different networks that need further inspection.

**Fig 2:**
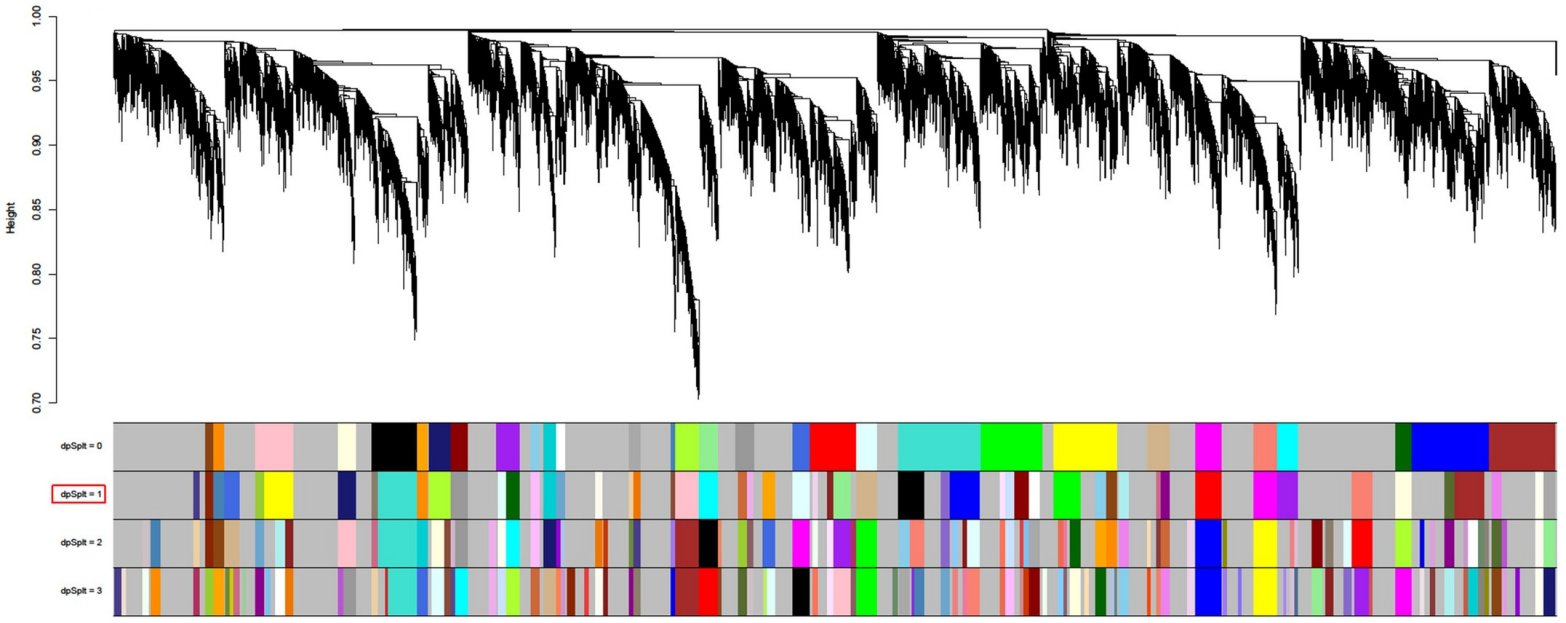
Dendrogram denoting modules in healthy network. Modules are illustrated with different colors obtained with different module detection sensitivity parameter called *deepSplit*. Each row with colored set of modules is detected with a certain *deepSplit* (between 0 and 3). The number of modules and average size are: *deepSplit* 0 (modules: 31, average size: 379.58) *deepSplit* 1 (modules: 55, average size: 235.05) *deepSplit* 2 (modules: 84, average size: 163.98) *deepSplit* 3 (modules: 105, average size: 137.3).

For further analysis, we cut the tree to generate modules (clusters) from the resulting dendrogram. A dynamic branch cutting method called “hybrid” is used to determine modules, which is implemented in *cutreeHybrid* function. Fig 2 shows resulting dendrogram for healthy samples with different cut-off levels corresponding to different sets of modules. Modules on the bottom of the figure are illustrated with different colors, and represent the branches of the clustering tree, which can be split by using *deepSplit* argument, which allows a rough control over sensitivity; we used following parameters: *deepSplit* = 1, cut height = 0.99, and minimum module size = 27. This parameter setting resulted in 55 modules with average size 235. Finally, module eigengenes are calculated, which provides quantitative assessments in further analysis.

### Comparing the modules between healthy and T1D networks

**Fig 3:**
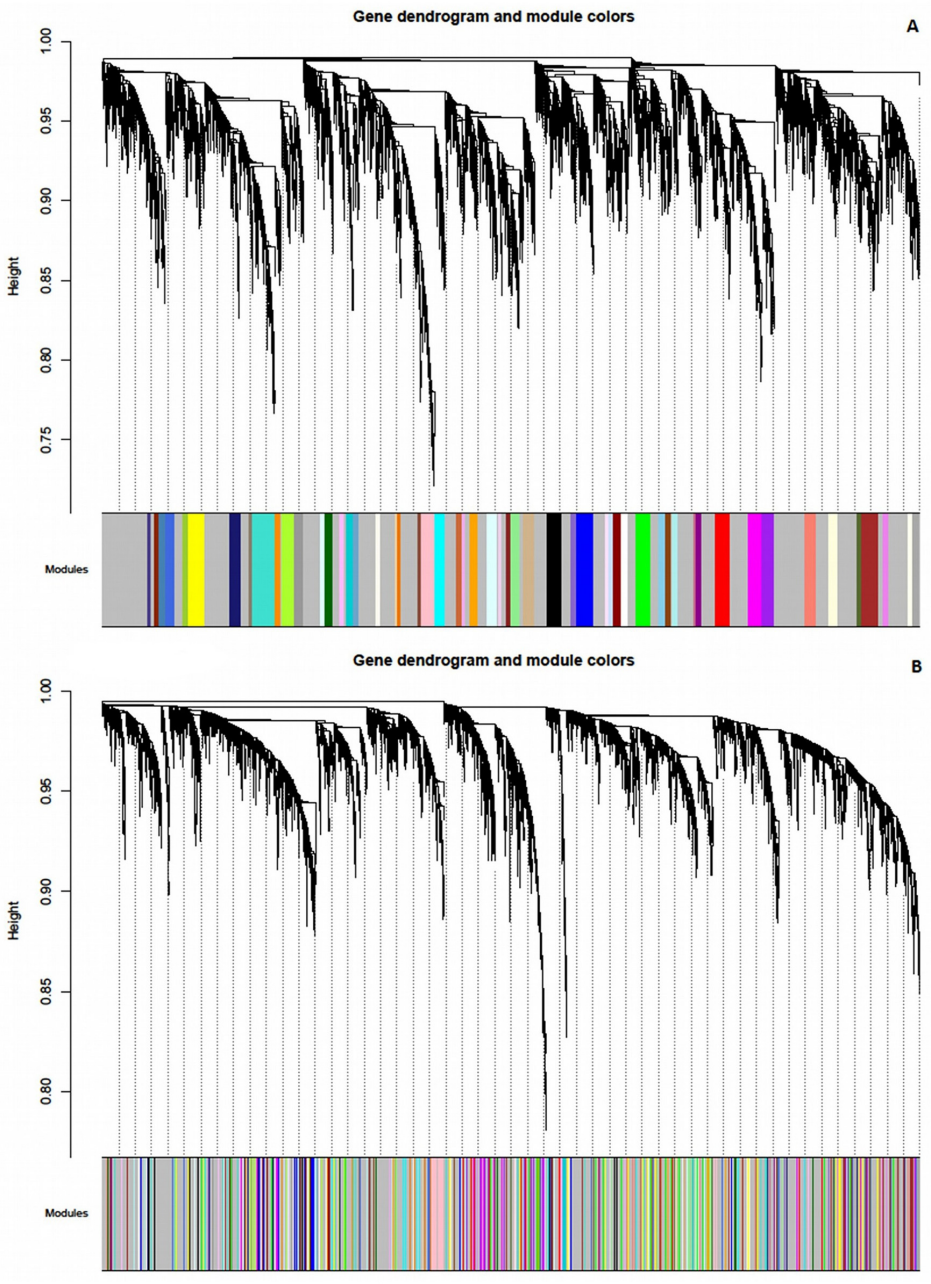
Visual representation of the changes in the module structure between (A) healthy network and (B) T1D network.

After generating 55 modules from network, based on healthy samples, we compared the results with the corresponding set of modules in T1D network. Initially, visual representation was done, to obtain a general idea of how modular structure changes between networks. In Fig 3, the same colors that are assigned to the genes based on the module membership in healthy network (Fig 3A) are applied to the corresponding genes in the T1D network (Fig 3B). Few modules are preserved, but there are considerable changes between the two networks which we further investigated using a quantitative way of assessing module preservation.

In contrast to the idea of the original paper which proposes identifying modules with strong evidence of preservation between reference and test network [28], we aimed to identify modules that are weakly preserved between networks (see Materials and Methods). We hypothesized that modules that are weakly preserved in T1D may highlight dysregulated pathways in disease that were acquired or lost when compared to a healthy network. Table 1 lists identified modules, along with their size and *Z*_*summary*_ values. Modules with *Z*_*summary*_< 5 are considered to have low preservation, which is the cut-off used to select modules for further analysis. Following modules are selected based on that criterion: Royalblue, Navajowhite, Yellowgreen and Bisque. Grey and Gold modules are excluded from analysis, as these are artificial modules containing all genes that were not assigned to any module.

**Table 1:** Resulting modules in healthy network compared to modules in T1D network.

Module name	Size	*Z*_*summary*_	Module name	Size	*Z*_*summary*_
pink	166	36,80	mediumpurple3	61	11,77
darkgrey	87	32,74	lightyellow	113	11,35
brown	208	29,29	darkmagenta	64	10,99
purple	150	28,85	lightcyan1	60	10,96
lightgreen	118	28,03	cyan	128	10,88
darkturquoise	90	27,26	palevioletred3	31	10,56
darkred	101	27,06	green	187	10,52
blue	209	24,84	darkorange	78	10,40
ivory	55	23,61	orange	86	10,21
magenta	160	23,33	salmon4	33	9,74
red	185	21,98	thistle2	39	9,37
lightcyan	119	21,16	yellow	198	8,60
orangered4	61	20,54	sienna3	64	8,13
steelblue	73	18,98	paleturquoise	68	8,02
white	78	16,94	skyblue	77	7,89
greenyellow	150	16,72	floralwhite	55	7,46
grey60	119	16,50	darkgreen	96	7,45
tan	144	16,13	thistle1	39	7,43
darkolivegreen	64	15,35	saddlebrown	75	6,95
lightsteelblue1	61	14,34	black	180	5,68
darkslateblue	42	13,99	plum2	41	5,27
salmon	141	13,82	turquoise	269	5,10
plum1	62	13,09	bisque	42	4,97
violet	67	12,79	yellowgreen	64	3,91
brown4	45	12,69	grey	400	2,84
darkorange2	50	12,54	navajowhite	30	2,62
skyblue3	63	12,52	gold	100	2,12
midnightblue	127	12,11	royalblue	108	1,12

### Module enrichment analysis

Pathway enrichment analysis of the interesting modules was performed with ConsensusPathDB [30] and EnrichNet [29] and results are shown for Royalblue module. Table 2 shows functionally enriched pathways obtained from ConsensusPathDB by setting q-value < 0.05. Results of pathway enrichment analysis obtained from EnrichNet are presented in Table 3, where XD-score denotes significance of the enriched pathway. Findings with higher scores are more significant than low-scoring results. Only significant hits with overlap size ≥ 2 (genes that are overlapping in the same pathway) were considered.

**Table 2:** Enrichment results from ConsensusPathDB.

KEGG ID	Pathway	Count	p-value	Genes
hsa04932	Non-alcoholic fatty liver disease (NAFLD)	8	2.17E-07	NDUFA4; BCL2L11; PIK3CA; FAS; IL1B; SDHB; UQCRB; CXCL8
hsa05010	Alzheimer´s disease	6	7.60E-05	NDUFA4; FAS; ADAM17; IL1B; SDHB; UQCRB
hsa05142	Chagas disease	4	9.99E-04	PIK3CA; IL1B; CXCL8; FAS
hsa04668	TNF signaling pathway	4	1.23E-03	TAB3; PIK3CA; IL1B; FAS
hsa04621	NOD-like receptor signaling pathway	3	1.84E-03	TAB3; CXCL8; IL1B
hsa05162	Measles	4	2.54E-03	RAB9A; PIK3CA; IL1B; FAS
hsa04210	Apoptosis	3	5.90E-03	PIK3CA; IL1B; FAS
hsa05164	Influenza A	4	6.57E-03	PIK3CA; IL1B; CXCL8; FAS
hsa04064	NF-kappa B signaling pathway	3	6.90E-03	TAB3; CXCL8; IL1B
hsa05016	Huntington´s disease	4	9.22E-03	NDUFA4; SDHB; UQCRB; DCTN4
hsa05143	African trypanosomiasis	2	9.25E-03	FAS; IL1B
hsa04620	Toll-like receptor signaling pathway	3	1.05E-02	PIK3CA; IL1B; CXCL8
hsa05146	Amoebiasis	3	1.13E-02	CXCL8; IL1B; PIK3CA
hsa05332	Graft-versus-host disease	2	1.33E-02	FAS; IL1B
hsa04940	Type I diabetes mellitus	2	1.45E-02	FAS; IL1B

Table shows resulting KEGG pathways enriched in Royalblue module.

**Table 3:** Enrichment results from EnrichNet.

KEGG ID	Description	XD-score	q-value	Count	Genes
hsa05332	Graft-versus-host disease	0.60	0.32	2	FAS; IL1B
hsa04940	Type I diabetes mellitus	0.57	0.32	2	FAS; IL1B

Table shows resulting KEGG pathways enriched in Royalblue module.

Both ConsensusPathDB and EnrichNet identified KEGG pathway “Type I diabetes mellitus” as functionally enriched in Royalblue module. Genes that are identified to be part of this pathway are FAS and IL1B. Another significantly enriched pathway identified by both tools (Tables 2 and 3) is “Graft-versus-host disease”, an immune-mediated disease that also involves FAS and IL1B [35]. Examples of other pathways identified by ConsensusPathDB only (Table 2) are “NOD-like receptor signaling pathway” and “Toll-like receptor signaling pathway”. These pathways are known to be key components in the innate immune system that may promote process leading to autoimmune diabetes [36,37].

### Topological analysis with Betweenness centrality measure

Topological analysis of the modules obtained from WGCNA was focused on the betweenness centrality (*BC*) of the genes within the modules. Since this measure reflects influence over the “information transfer” between different nodes (genes), we identified genes for which betweenness is considerably changed between the two networks (healthy/T1D). *BC* values for the genes in Royalblue module are presented in Table 4.

**Table 4:** Betweenness centrality (*BC*) ranks for genes belonging to Royalblue module (in both healthy and T1D network).

Gene ID	*BC* (healthy)	Gene ID	*BC* (healthy)	Gene ID	*BC* (T1D)	Gene ID	*BC* (T1D)
DDX52	2658.15	PAPD4	8.90	NUCKS1	1143.37	NBN	58.76
NUCKS1	2377.35	WDR61	8.36	ANKRD10-IT1	1087.81	BTF3	46.35
SNX14	1598.20	RAB9A	5.29	NDUFA4	757.67	CCNC	45.67
HNRNPH1	1186.69	BTF3	0.24	ANP32E	747.13	DCTN4	41.05
ANP32A	996.50	BORA	2.51	TNPO3	676.32	TMEFF2	40.64
ANKRD10-IT1	970.57	PEX2	0.50	TAB3	610.91	ZFX	29.52
ASB3	662.10	UGGT1	0.00	PAPD4	533.85	SNX14	29.13
IL1B	518.17	WDR48	0.00	CCRN4L	526.38	NRIP1	16.33
SMC3	334.62	PRDM1	0.00	SMC3	441.31	FAS	8.77
RUFY2	332.00	CXCL8	0.00	RIT1	433.20	PGGT1B	1.16
MRPL44	287.12	PYROXD1	0.00	HNRNPH1	385.24	TSN	0.90
TMEFF2	168.00	MED13L	0.00	EIF4B	381.40	MRPL44	0.33
SDHB	168.00	MIS18BP1	0.00	RSL24D1	358.21	HNRNPA1	0.00
UQCRB	131.03	SCAF4	0.00	SCAF4	332.77	CEP350	0.00
EIF4B	117.96	ATXN2L	0.00	ADAM17	332.43	UQCRB	0.00
PGGT1B	105.05	KCNJ14	0.00	STX2	320.00	LAPTM4B	0.00
TAB3	96.27	CCNC	0.00	PEX2	280.63	MED13L	0.00
HNRNPA1	94.58	RIMKLB	0.00	THUMPD3	277.68	RAB9A	0.00
THUMPD3	80.77	YIPF6	0.00	CXCL8	263.80	SBNO1	0.00
FAS	71.69	RIT1	0.00	RAB21	207.27	EMC7	0.00
NBN	43.12	NSG1	0.00	IL1B	185.50	CHEK1	0.00
STX2	36.15	LARP7	0.00	KCNJ14	162.00	BORA	0.00
NRIP1	32.52	BCL2L11	0.00	YIPF6	161.15	DDX52	0.00
HAUS2	27.29	ATXN7	0.00	TAF9	153.78	PYROXD1	0.00
UFM1	27.29	LAPTM4B	0.00	ATXN7	142.64	ATXN2L	0.00
NDUFA4	19.4	SLC4A4	0.00	MIS18BP1	138.98	WDR48	0.00
TPP2	18.17	CHEK1	0.00	ANP32A	126.32	RIMKLB	0.00
MTRR	16.28	ADAM17	0.00	CTSB	113.57		
ANP32E	12.19	TAF9	0.00	MBTD1	108.41		
RAB21	11.92	CEP350	0.00	TPP2	69.29		

**Fig 4:**
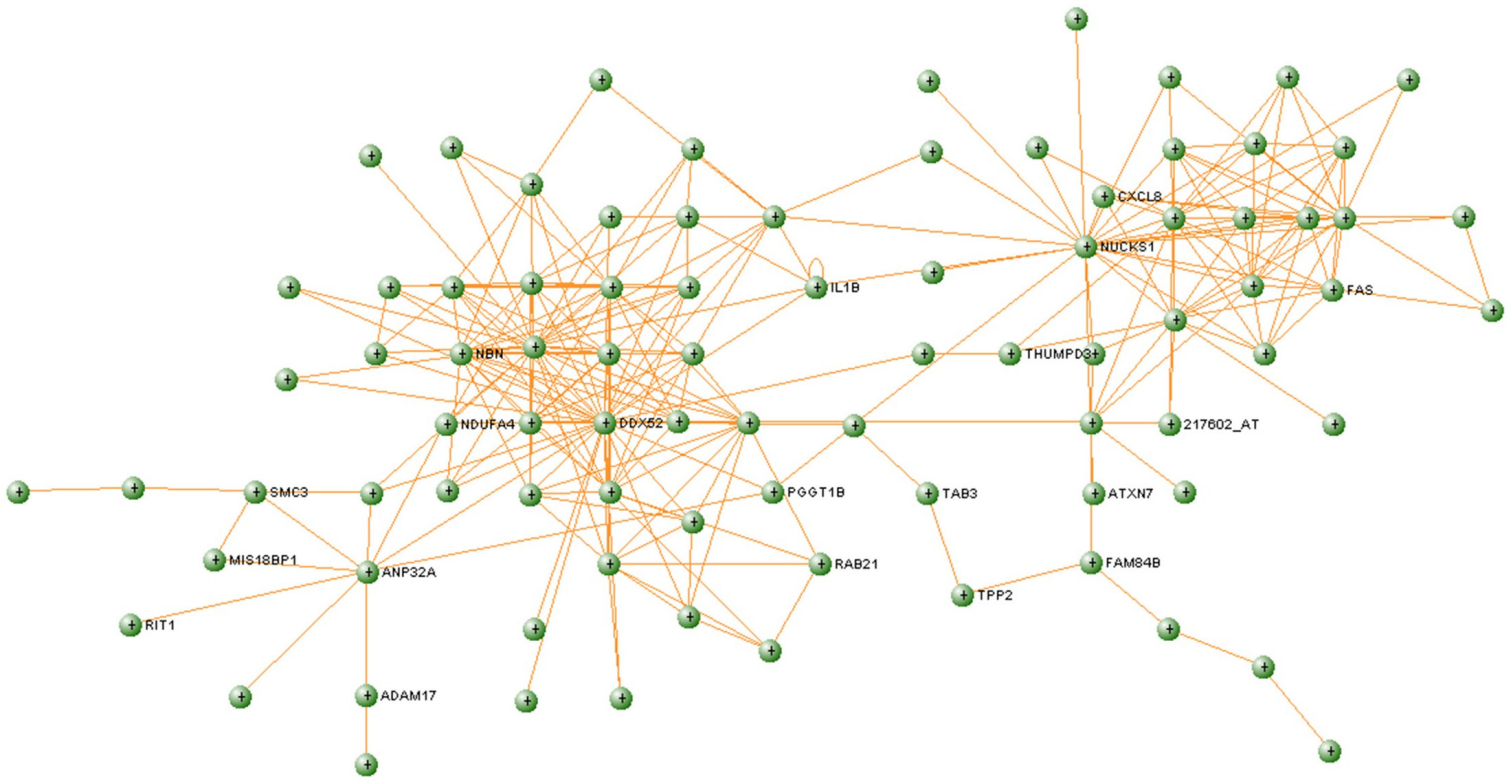
Royalblue module extracted from the healthy network.

**Fig 5:**
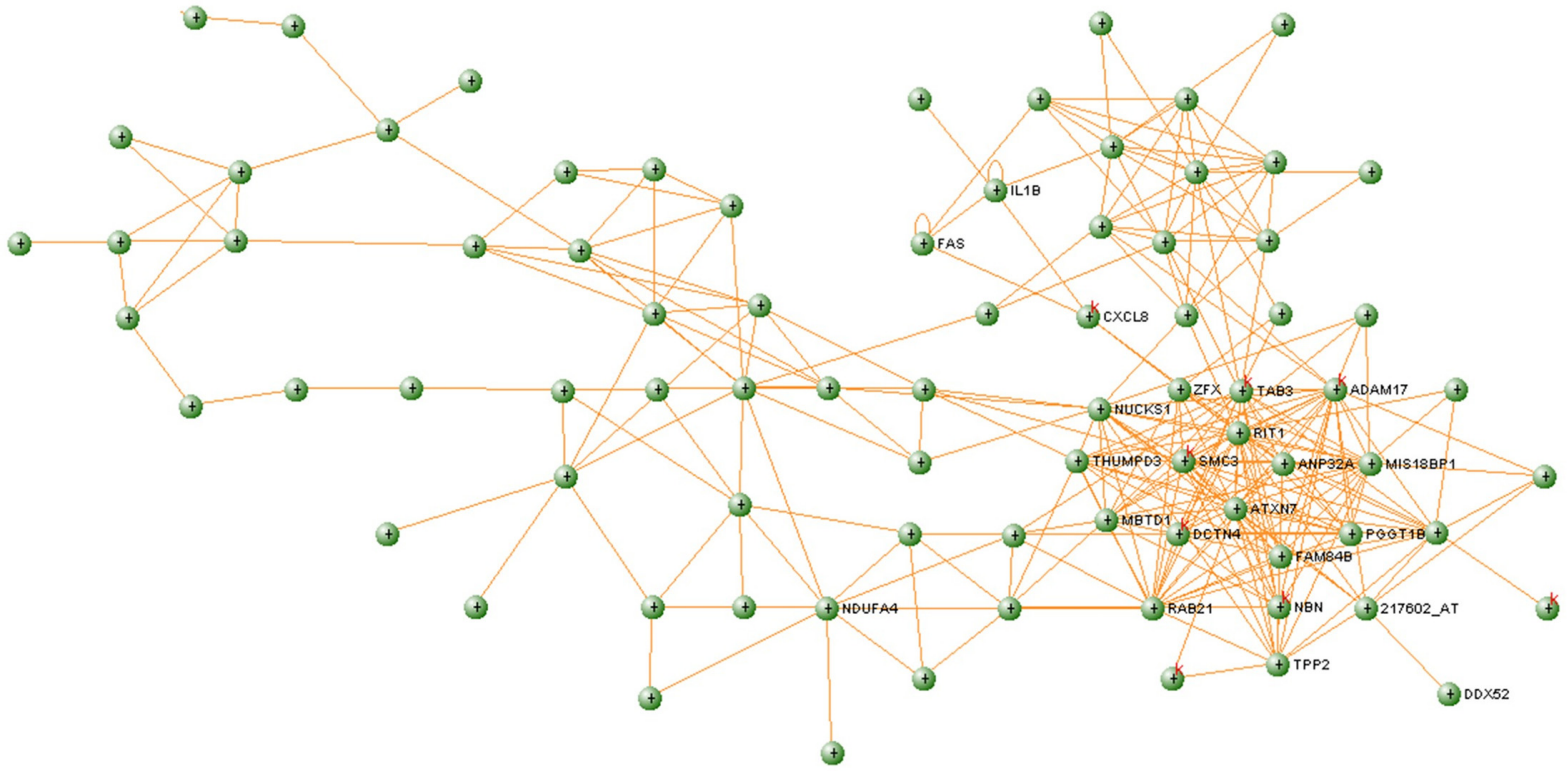
Royalblue module extracted from the T1D network.

Using betweenness value to rank genes in the healthy network, we identified DDX52, as the gene with highest betweenness (*BC*_healthy_ = 2 658.15), suggesting that it has a central role in information transfer in this module. However, the DDX52 gene in T1D network shows an aberrant structure, with only one connection and *BC*_T1D_ = 0. This gene is part of the DEAD box helicases protein family [38] which functions to separate the strands of short mRNA duplexes. Several of the proteins in this family, such as DDX3 and DDX42, are related to regulation of the immune activity [39]. DDX52 has also been shown to be under-expressed in relation to the immune response in another T1D study [40]. In this study, DDX52 plays an important topological role in the healthy network since it is in the center of one of the most interconnected regions (Fig 4). However, in the diabetes network, DDX52 only interacts with one direct neighbor (Fig 5). The function of DDX52 in T1D is still not known, but due to these major differences in the topology involving this protein, it is worth investigating whether this could be one of the factors that is aberrant at early stage at development of T1D.

NDUFA4 is a contrasting example of the gene with a large difference in *BC* value changing from being low-betweenness gene in healthy to high-betweenness gene in T1D network (Table 4). NDUFA4 encodes a subunit of the complex 1 of the mitochondrial electron transport chain with NADH dehydrogenase and oxidoreductase functions. The role of this gene is not known in T1D, but it is found to be over-expressed in T1D compared to controls and pre-T1D patients [41].

Another gene with the betweenness centrality that largely deviates between healthy and T1D network is SNX14 (sorting nexin 14). The role of this gene in T1D is not known, but there is one previous study showing SNX14 is significantly down-regulated (FDR<0.05) in children that progressed to T1D [42].

Betweenness centrality analysis of the rest of selected modules: Navajowhite, Yellowgreen, and Bisque (S1–S3 Tables) revealed a number of genes with known or potential role in T1D. The gene with the highest *BC* value in Navajowhite disease module is CAPRIN1 (S1 Table), which encodes proteins involved in synaptic plasticity in neurons and cell proliferation and migration [43]. This gene has been associated with autoimmune diseases in mouse [44], but so far the similar role is yet to be confirmed in human autoimmune diseases. In Yellowgreen module, SCAF11 (SIP-1) shows high *BC* in healthy and very low *BC* in T1D (S2 Table). SCAF11 is known to be involved in Behcet´s disease [45] that has some features of autoimmunity. Furthermore, another gene identified according to the same criteria is ITFG1 (or TIP), which modulates T-cell function and has protective effect in graft-versus-host disease model [46]. In the last module we analyzed (Bisque) there were also several interesting genes that show large difference between *BC* values in healthy versus T1D module (S3 Table). IL1A or IL-1 (*BC*_T1D_ = 60.7; *BC*_healthy_ = 0) is a pro-inflammatory cytokine that takes part in the “diabetes type I pathway”.

## Discussion

Network-based analysis provides higher level connections between molecules and their involvement in different pathways, which is a good starting point for investigating complex diseases, such as T1D. The present study focuses on co-expression module-based analysis using WGCNA in combination with other network topology information. The results of the study reveal biological pathways that are enriched in co-expression modules and show aberrant structure in T1D network compared to the corresponding modules in co-expression network of healthy controls.

Five modules were chosen for further analysis, based on the loss of preservation, as explained in the result section. The results of the analysis of top-ranked Royalblue module will be discussed here in detail. Lists of the enriched terms in other modules can be found in S4–S7 Tables. Functional enrichment analysis of this module was performed with EnrichNet and ConsensusPathDB. EnrichNet utilizes information from the molecular network structure connecting two gene/protein sets to score distances between input set of genes and pathways in a reference database. EnrichNet tries to overcome some limitations of the traditional over-representation based enrichment analysis, by calculating XD-score, which is relative to the average distance to all pathways in a reference database. The analysis resulted in several pathways and processes with a clear connection to different mechanisms that are associated to T1D. One of the KEGG pathways that showed enrichment was “Type I diabetes mellitus” and the analyzed module contains two genes from this pathway—FAS and IL1B. FAS belongs to the TNF-receptors superfamily and plays a major role in the programmed cell death. Apoptosis mediated by FAS appears to be the main mechanism in T cell-mediated damage to insulin-producing beta cells [47]. IL1B is a cytokine that serves as an important mediator in the inflammatory process and is also part of the main mechanisms of beta cell death in diabetes [48]. Among other pathways that are significantly enriched (Table 2), we found “Non-alcoholic fatty liver disease (NAFLD)”, “TNF-signaling pathway”, “NOD-like receptor signaling pathway” etc.

**Fig 6:**
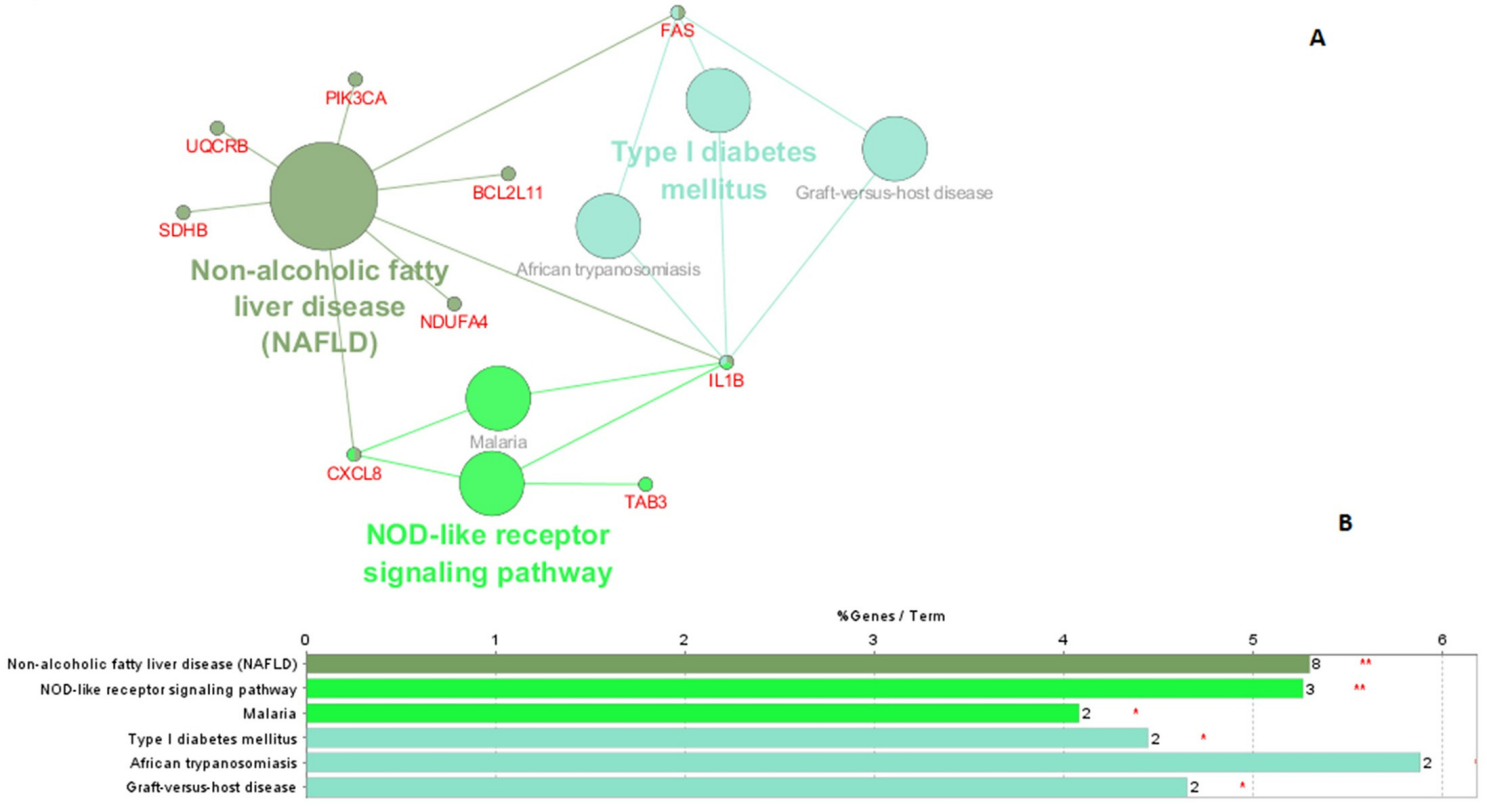
Functionally enriched KEGG pathways identified by ClueGO. (A) The size of the nodes reflects significance of the term. Network includes genes that are shared between different KEGG pathways (B) Chart represents significant KEGG pathways where bars show number of genes associated to each term. Level of significance for terms is marked using 1) **; if the term is over-significant (p-value<0.001), 2) *; if the terms is significant (0.001<p-value<0.05), and 2). (dot); 0.05<p-value<0.01).

To further understand the interplay between significant pathways within the module and identify which genes they have in common, we used ClueGO (http://apps.cytoscape.org/apps/cluego/) [49]. Resulting network (Fig 6) illustrates network of six significantly enriched KEGG pathways along with the genes that are shared between these pathways (Fig 6A). Fig 6B illustrates significant KEGG pathways where bars show number of genes associated to each term. There are three genes shared by several pathways—IL1B (six pathways), FAS (four pathways), and CXCL8 (three pathways). This also confirms the relevance of the derived modules for identifying key players in T1D. Interestingly, analysis with VisANT confirms that these three genes are connected in a T1D network generated from the Royalblue module, while there is no such connection in the corresponding healthy module. CXCL8 provides the main connection between FAS—IL1B and the main cluster (the most interconnected region) in the T1D module (Fig 5), containing genes related to the immune system. In corresponding healthy module (Fig 4), these direct connections between CXCL8, FAS and IL1B are absent. CXCL8 (also known as IL8) produces interleukin 8, a chemokine which plays an important role in the inflammatory response and it is produced by many cell types [50]. In addition, high levels of interleukin 8 have been found in the saliva [51] and in the blood [38] of children with T1D. Due to the position of this gene in the diabetes network (Fig 5) and the confirmation of its increased expression levels in diabetic patients, interleukin 8 s may play an important part in the development of diabetes.

Another interesting gene that is part of the tightly connected cluster in T1D module (Fig 5) is ADAM17, which encodes the tumor necrosis factor-alpha converting enzyme (TNFR). ADAM17 plays a central role in cell regulation and thus it is related to many diseases, including T1D [52]. Earlier studies indicated that intermembrane activity of ADAM17 is possible factor that influences concentrations of TNFRs in blood in T1D patients [53,54].

There are other interesting genes that build the tightly interconnected cluster in Fig 5. We found genes that are related to cancer (NBN, FAM84B), Huntington's disease (DCTN4), Parkinson's disease (NUCKS1), Alzheimer's disease (SMC3), amongst others. Parkinson's disease is an autoimmune disease [55], and recent findings support the theory that Alzheimer's also appears to have an autoimmune component to the disease [56]. Previous work on Huntington's disease showed an activation of the immune system and altered immune response before the manifestation of clinical symptoms [57]. In summary, the part of the identified module aberrant in T1D may give the indication of the process shared amongst the above mentioned diseases. This result agrees with the enrichment analysis that found KEGG pathways “Alzheimer´s disease” and “Huntington´s disease” functionally enriched (Table 2). Further analysis of the dense cluster identified in the disease module (Fig 5) reveals another gene, TAB3, a part of “NF-kappa B signaling pathway” that is associated with the activation of the immune systems, particularly in response to external factors, such as inflammation. This pathway is found functionally enriched in Royalblue module (Table 2) and it was recently proposed that this pathway have important implications on the development of novel therapeutic strategies for T1D [58]. Since several of the genes that build the densely connected cluster in the Royalblue module are related to the immune diseases, it is of great interest to further investigate other genes in that cluster (i.e. ATXN7, PGGT1B, MIS18BP1) that have not been previously associated with T1D, and may contribute to extending our knowledge about the disease.

Topological analysis based on betweenness centrality measure (*BC*) revealed some high-betweenness proteins that may act as important links between different pathways. One of the genes identified based on betweenness centrality criteria that may play a role in T1D is NDUFA4. This finding, together with the results from the enrichment analysis, highlights NDUFA4 as part of three significantly enriched pathways (Table 2) with potential connection to diabetes, and identifies this gene as an interesting candidate for further investigation. One of these pathways is “Non-alcoholic fatty liver disease (NAFLD)”, which has been linked to T1D in children in previous studies [59,60].

Another interesting gene that shows a large difference between *BC* in healthy versus T1D Bisque module (see Results) is IL1A or IL-1, a pro-inflammatory cytokine that takes part in the “diabetes type I pathway”. The inhibition of IL-1 action has clinical efficacy in several inflammatory diseases including hereditary auto-inflammatory disorders and type 2 diabetes mellitus. [61]. Due to its modulating effect on the interaction between the innate and adaptive immune systems, IL-1 has suggested has been evaluated as a potential target in the autoimmune diabetes mellitus [61]. This finding also confirms that the approach we propose here offers insights into pathways and genes with known involvement in T1D and may serve as a good starting point for identifying novel mechanisms. SH2B2 is another gene in the same module which is a part of “insulin signaling pathway”, but its role in T1D is not known.

Apart from focusing on modules with low preservation between healthy and T1D network, we contrasted our results by analyzing the most preserved module (Pink module), and investigated which role this module may have in the disease. The resulting list of the pathways that are enriched in Pink module with p-value<0.05 are: “HTLV-I infection”, “T cell receptor signaling pathway”, and “Changas disease” (S8 Table). T cell receptor signaling pathway is related to the immune system and known to be associated to T1D. There are two gene members in this module that account for the enrichment in this pathway: DLG1 and NFATC3. DLG1 is involved in signal transduction, cell proliferation, and synaptogenesis, which are important functions that seem to be preserved when comparing heathy and disease module. Mutations in DLG1 are known to be associated to Chrohn´s disease [62], which is an immune related disease. NFATC3 has a crucial role in the development of the immune system and T-cell development [63]. This finding suggests that the highly preserved module in this case encloses functions that are important for the immune system and also shows preserved density and connectivity pattern between healthy and disease module.

In summary, our results show that the application of WGCNA, along with topological analysis and functional enrichment can be used to detect modules associated with T1D in children. Detected modules are utilized for exploratory analysis of dysregulated pathways in disease, as exemplified by the identified pathways such as “Type I diabetes mellitus”, Non-alcoholic fatty liver disease (NAFLD)”, “NOD-like receptor signaling pathway”. In addition, the approach we report here may help identifying candidate genes that are likely to be associated with the disease, such as IL-1, that was found to be significantly increased in newly diagnosed T1D patients [64]. Other identified genes that confirm the relevance of this approach are IL1B, FAS, CXCL8 etc. Previous work on network analysis in T1D was manly focused on using protein-protein interaction (PPI) interaction networks to find candidate genes in disease [65]. While other studies were focused on specific regions of interest for T1D, such as Major Histocompatibility Complex (MHC) region on chromosome 6p21 [66], our study identifies global network structure that allows us to explore key pathways and candidate genes from T1D co-expression modules derived from whole transcriptome. This work highlights the importance of the systems biology approach to study complex disease by analyzing the inherent modularity of the T1D co-expression network. Future efforts should be made to further investigate system-level properties of the modules associated to T1D, as well as other topological properties of the genes involved in identified modules.

## Supporting Information

S1 Table Betweenness centrality (BC) ranks for genes belonging to Navajowhite module. (DOC)

S2 Table Betweenness centrality (BC) ranks for genes belonging to Yellowgreen module. (DOC)

S3 Table Betweenness centrality (BC) ranks for genes belonging to Bisque module. (DOC)

S4 Table Function enrichment results for Navajowhite module. Table shows GO biological process and molecular function for gene members of Navajowhite module (p-values<0.05), gene count>2.
(DOC)

S5 Table Function enrichment results for Yellowgreen module. Table shows GO biological process and molecular function for gene members of Yellowgreen module (p-values<0.05), gene count>2.
(DOC)

S6 Table Function enrichment results for Bisque module. Table shows GO biological process and molecular function for gene members of Bisque module (p-values<0.05), gene count>2.
(DOC)

S7 Table Pathway enrichment results for Bisque module. Table shows resulting KEGG pathways enriched in Bisque module.
(DOC)

S8 Table Pathway enrichment results for Pink module. Table shows resulting KEGG pathways enriched in Pink module.
(DOC)
