# Supplementary material for: Gene Co-Expression Network Analysis for Identifying Modules and Functionally Enriched Pathways in Type 1 Diabetes
Source: PLoS One. 2016 Jun 3;11(6):e0156006. doi: 10.1371/journal.pone.0156006 (PMC4892488; doi:10.1371/journal.pone.0156006)
Supplement: S1 Table — (DOC) [file pone.0156006.s001.doc]

S1 Table. Betweenness centrality (*BC*) ranks for genes belonging to Navajowhite module.

| Gene ID | | | *BC* (healthy) | Gene ID | *BC* (T1D) |  |
| --- | --- | --- | --- | --- | --- | --- |
|  | SLC4A7 | 35.99 | | CAPRIN1 | 23.64 | |
|  | SEPT2 | 35.99 | | MGEA5 | 23.44 | |
|  | KLF12 | 35.99 | | TMEM63A | 15.81 | |
|  | MGEA5 | 35.99 | | CUL2 | 14.96 | |
|  | SETD6 | 35.99 | | SLC25A16 | 14.87 | |
|  | TCF4 | 32.34 | | TCF4 | 14.87 | |
|  | AQR | 4.15 | | FLJ42627 | 8.26 | |
|  | RARA | 2.84 | | UBFD1 | 6.16 | |
|  | MED13L | 0.98 | | WIPF2 | 5.92 | |
|  | CUL2 | 0.87 | | RARA | 5.02 | |
|  | SLC25A16 | 0.68 | | SPATA5L1 | 4.87 | |
|  | TMEM63A | 0.30 | | KLF12 | 4.87 | |
|  | HOXA13 | 0.14 | | SEPT2 | 4.13 | |
|  | CAPRIN1 | 0.00 | | SLC4A7 | 3.33 | |
|  | FLJ42627 | 0.00 | | MED13L | 2.69 | |
|  | UBFD1 | 0.00 | | AQR | 1.18 | |
|  | SPATA5L1 | 0.00 | | HOXA13 | 0.94 | |
|  | SLA | 0.00 | | SLA | 0.67 | |
|  | WIPF2 | 0.00 | | SETD6 | 0.66 | |
