# Supplementary material for: Gene Co-Expression Network Analysis for Identifying Modules and Functionally Enriched Pathways in Type 1 Diabetes
Source: PLoS One. 2016 Jun 3;11(6):e0156006. doi: 10.1371/journal.pone.0156006 (PMC4892488; doi:10.1371/journal.pone.0156006)
Supplement: S2 Table — (DOC) [file pone.0156006.s002.doc]

S2 Table. Betweenness centrality (*BC*) ranks for genes belonging to Yellowgreen module.

| Gene ID | *BC* (healthy) | Gene ID | *BC* (T1D) |
| --- | --- | --- | --- |
| BTN2A1 | 1 190.12 | SRRM2 | 322.08 |
| SRRM2 | 702.76 | BTN2A1 | 321.06 |
| SCAF11 | 626.03 | TAF1D | 318.00 |
| PLCG1 | 137.08 | RBM5 | 256.08 |
| KLHL3 | 113.74 | LGALS8 | 220.30 |
| TTN | 112.00 | KBTBD2 | 216.00 |
| AFF4 | 24.29 | CCDC69 | 184.45 |
| TAF1D | 19.04 | ITFG1 | 154.12 |
| HNRNPUL1 | 12.05 | ITSN2 | 110.53 |
| LGALS8 | 10.01 | SLC17A5 | 110.00 |
| RBM5 | 9.23 | RGCC | 110.00 |
| SESN1 | 2.83 | TTN | 82.66 |
| SEPT9 | 2.74 | VAPB | 68.26 |
| IST1 | 1.72 | AFF4 | 60.71 |
| TRAPPC10 | 1.39 | PLCG1 | 33.66 |
| SCML1 | 1.13 | USP20 | 32.30 |
| LUC7L3 | 1.07 | SEPT9 | 31.97 |
| SLC17A5 | 0.86 | KLHL3 | 28.43 |
| PHF2 | 0.40 | HNRNPUL1 | 20.40 |
| ZNF266 | 0.17 | SCAF11 | 17.21 |
| ZNF789 | 0.00 | LCOR | 14.74 |
| CCDC69 | 0.00 | SNRNP200 | 12.23 |
| SNX5 | 0.00 | TRAPPC10 | 6.49 |
| ITFG1 | 0.00 | FZD3 | 5.07 |
| SIK3 | 0.00 | FOXK1 | 4.82 |
| TUG1 | 0.00 | TUG1 | 4.63 |
| ITSN2 | 0.00 | ASXL1 | 2.20 |
| LCOR | 0.00 | MRPS11 | 2.00 |
| ASXL1 | 0.00 | CASC3 | 1.33 |
| CASC3 | 0.00 | PHF2 | 1.03 |
| KBTBD2 | 0.00 | SESN1 | 0.33 |
| MRPS11 | 0.00 | ZNF510 | 0.33 |
| SNRNP200 | 0.00 | SCML1 | 0.00 |
| BSDC1 | 0.00 | MED31 | 0.00 |
| FOXK1 | 0.00 | BSDC1 | 0.00 |
| MED31 | 0.00 | SALL2 | 0.00 |
| VAPB | 0.00 | ZNF789 | 0.00 |
| FZD3 | 0.00 | IST1 | 0.00 |
| ZNF510 | 0.00 | ZNF266 | 0.00 |
| RGCC | 0.00 | LUC7L3 | 0.00 |
|  |  |  |  |
