# Supplementary material for: Gene Co-Expression Network Analysis for Identifying Modules and Functionally Enriched Pathways in Type 1 Diabetes
Source: PLoS One. 2016 Jun 3;11(6):e0156006. doi: 10.1371/journal.pone.0156006 (PMC4892488; doi:10.1371/journal.pone.0156006)
Supplement: S3 Table — (DOC) [file pone.0156006.s003.doc]

S3 Table. Betweenness centrality (*BC*) ranks for genes belonging to Bisque module.

| Gene ID | *BC* (healthy) | Gene ID | *BC* (T1D) |
| --- | --- | --- | --- |
| BAZ2A | 139.42 | FKSG49 | 182.87 |
| FKSG49 | 139.42 | DYNC1LI2 | 130.68 |
| KIAA0226L | 139.42 | KIAA0226L | 101.76 |
| ZNF395 | 139.42 | SLC25A36 | 93.58 |
| LRP8 | 117.09 | IL1A | 60.74 |
| ZNF136 | 73.72 | APOPT1 | 57.58 |
| SLC25A36 | 8.17 | DTX4 | 54.08 |
| CADM1 | 8.13 | WIPI2 | 48.83 |
| RPS28 | 7.63 | BAZ2A | 46.13 |
| WIPI2 | 0.97 | FXYD3 | 43.54 |
| ZNF362 | 0.67 | SH2B2 | 42.17 |
| RB1CC1 | 0.44 | RPS28 | 39.43 |
| SNX9 | 0.32 | TROVE2 | 29.28 |
| OSBPL2 | 0.25 | LRP8 | 28.60 |
| ERF | 0.22 | ZNF136 | 25.17 |
| SH2B2 | 0.20 | GOSR2 | 24.60 |
| FXYD3 | 0.18 | TNPO2 | 22.81 |
| GOSR2 | 0.14 | OSBPL2 | 19.29 |
| DYNC1LI2 | 0.14 | PIK3R2 | 18.21 |
| TNPO2 | 0.00 | SNX9 | 17.57 |
| SIN3B | 0.00 | ZNF395 | 14.23 |
| TROVE2 | 0.00 | POM121L2 | 9.80 |
| DTX4 | 0.00 | FNBP1 | 9.59 |
| PIK3R2 | 0.00 | ZNF362 | 7.61 |
| CASZ1 | 0.00 | RB1CC1 | 3.63 |
| FNBP1 | 0.00 | CASZ1 | 3.06 |
| SWAP70 | 0.00 | C6orf106 | 2.74 |
| APOPT1 | 0.00 | CADM1 | 0.44 |
| IL1A | 0.00 | SIN3B | 0.24 |
| C6orf106 | 0.00 | SWAP70 | 0.00 |
| POM121L2 | 0.00 | ERF | 0.00 |
| YES1 | 0.00 | YES1 | 0.00 |
|  |  |  |  |
