# Supplementary material for: Gene Co-Expression Network Analysis for Identifying Modules and Functionally Enriched Pathways in Type 1 Diabetes
Source: PLoS One. 2016 Jun 3;11(6):e0156006. doi: 10.1371/journal.pone.0156006 (PMC4892488; doi:10.1371/journal.pone.0156006)
Supplement: S4 Table — Table shows GO biological process and molecular function for gene members of Navajowhite module (p-values<0.05), gene count>2. (DOC) [file pone.0156006.s004.doc]

S4 Table. Function enrichment results for Navajowhite module.

| GO ID | Description | Count | p-value |
| --- | --- | --- | --- |
| GO:0003712 | transcription cofactor activity | 4 | 0.0014 |
| GO:0048468 | cell development | 6 | 0.0102 |
| GO:0007423 | sensory organ development | 3 | 0.0125 |
| GO:0008134 | transcription factor binding | 3 | 0.0125 |
| GO:0034641 | cellular nitrogen compound metabolic process | 11 | 0.0165 |
| GO:0048729 | tissue morphogenesis | 3 | 0.0194 |
| GO:0000981 | RNA polymerase II transcription factor activity, sequence-specific DNA binding | 3 | 0.0204 |
| GO:0044249 | cellular biosynthetic process | 10 | 0.0285 |
| GO:0008219 | cell death | 5 | 0.0339 |
| GO:0032989 | cellular component morphogenesis | 4 | 0.0402 |
|  |  |  |  |
