# Supplementary material for: Gene Co-Expression Network Analysis for Identifying Modules and Functionally Enriched Pathways in Type 1 Diabetes
Source: PLoS One. 2016 Jun 3;11(6):e0156006. doi: 10.1371/journal.pone.0156006 (PMC4892488; doi:10.1371/journal.pone.0156006)
Supplement: S5 Table — Table shows GO biological process and molecular function for gene members of Yellowgreen module (p-values<0.05), gene count>2. (DOC) [file pone.0156006.s005.doc]

S5 Table. Function enrichment results for Yellowgreen module.

| GO ID | Description | Count | p-value |
| --- | --- | --- | --- |
| GO:0044260 | macromolecule metabolic process | 28 | 3.97e-05 |
| GO:0006139 | nucleobase-containing compound metabolic process | 22 | 8.7e-05 |
| GO:0046483 | heterocycle metabolic process | 22 | 0.000146 |
| GO:0006725 | cellular aromatic compound metabolic process | 22 | 0.000151 |
| GO:0043170 | macromolecule metabolic process | 28 | 0.00022 |
| GO:1901360 | organic cyclic compound metabolic process | 22 | 0.000274 |
| GO:0022613 | ribonucleoprotein complex biogenesis | 5 | 0.000605 |
| GO:0034641 | cellular nitrogen compound metabolic process | 22 | 0.00075 |
| GO:0003676 | nucleic acid binding | 16 | 0.00157 |
| GO:0019222 | regulation of metabolic process | 20 | 0.0109 |
| GO:0043933 | macromolecular complex subunit organization | 10 | 0.148 |
| GO:0044249 | cellular biosynthetic process | 18 | 0.0149 |
| GO:1901576 | organic substance biosynthetic process | 18 | 0.0178 |
| GO:0019904 | protein domain specific binding | 4 | 0.0271 |
| GO:0009887 | organ morphogenesis | 5 | 0.0308 |
| GO:0032989 | cellular component morphogenesis | 6 | 0.0476 |
|  |  |  |  |
