# Supplementary material for: Gene Co-Expression Network Analysis for Identifying Modules and Functionally Enriched Pathways in Type 1 Diabetes
Source: PLoS One. 2016 Jun 3;11(6):e0156006. doi: 10.1371/journal.pone.0156006 (PMC4892488; doi:10.1371/journal.pone.0156006)
Supplement: S6 Table — Table shows GO biological process and molecular function for gene members of Bisque module (p-values<0.05), gene count>2. (DOC) [file pone.0156006.s006.doc]

S6 Table. Function enrichment results for Bisque module.

| GO ID | Description | Count | p-value |
| --- | --- | --- | --- |
| GO:0006810 | transport | 14 | 0.00265 |
| GO:0006996 | organelle organization | 12 | 0.00292 |
| GO:0044260 | cellular macromolecule metabolic process | 20 | 0.00318 |
| GO:0008104 | protein localization | 9 | 0.00535 |
| GO:0019222 | regulation of metabolic process | 17 | 0.00567 |
| GO:0006139 | nucleobase-containing compound metabolic process | 15 | 0.00682 |
| GO:0019899 | enzyme binding | 7 | 0.0087 |
| GO:1902589 | single-organism organelle organization | 9 | 0.00888 |
| GO:0046483 | heterocycle metabolic process | 15 | 0.00934 |
| GO:0050817 | coagulation | 4 | 0.00948 |
| GO:0043170 | macromolecule metabolic process | 20 | 0.00954 |
| GO:0006725 | cellular aromatic compound metabolic process | 15 | 0.00956 |
| GO:0044249 | cellular biosynthetic process | 15 | 0.0119 |
| GO:1901360 | organic cyclic compound metabolic process | 15 | 0.0137 |
| GO:1901576 | organic substance biosynthetic process | 15 | 0.014 |
| GO:0005102 | receptor binding | 6 | 0.0172 |
| GO:0009611 | response to wounding | 5 | 0.0179 |
| GO:0050878 | regulation of body fluid levels | 4 | 0.0208 |
| GO:0045184 | establishment of protein localization | 7 | 0.0212 |
| GO:0070727 | cellular macromolecule localization | 6 | 0.0223 |
| GO:0050794 | regulation of cellular process | 21 | 0.0224 |
| GO:0034641 | cellular nitrogen compound metabolic process | 15 | 0.0251 |
| GO:0002757 | immune response-activating signal transduction | 3 | 0.0351 |
| GO:0044802 | single-organism membrane organization | 3 | 0.0363 |
| GO:0019538 | protein metabolic process | 13 | 0.0401 |
| GO:0003712 | transcription cofactor activity | 3 | 0.0438 |
|  |  |  |  |
