# Supplementary material for: Gene Co-Expression Network Analysis for Identifying Modules and Functionally Enriched Pathways in Type 1 Diabetes
Source: PLoS One. 2016 Jun 3;11(6):e0156006. doi: 10.1371/journal.pone.0156006 (PMC4892488; doi:10.1371/journal.pone.0156006)
Supplement: S7 Table — Table shows resulting KEGG pathways enriched in Bisque module. (DOC) [file pone.0156006.s007.doc]

S7 Table. Pathway enrichment results for Bisque module.

| Source | Pathway | Count | p-value | Genes |
| --- | --- | --- | --- | --- |
| Wikipathways | Interleukin-11 Signaling Pathway | 2 | 0.00274 | PIK3R2; YES1 |
| Wikipathways | IL-1 signaling pathway | 2 | 0.00425 | PIK3R2; IL1A |
| Wikipathways | Kit receptor signaling pathway | 2 | 0.00488 | PIK3R2;SH2B2 |
| KEGG | Apoptosis | 2 | 0.0101 | PIK3R2; IL1A |
| Wikipathways | TCR Signaling Pathway | 2 | 0.0115 | PIK3R2; IL1A |
| Wikipathways | Senescence and Autophagy in Cancer | 2 | 0.0148 | IL1A; RB1CC1 |
| KEGG | Neurotrophin signaling pathway | 2 | 0.0191 | SH2B2; PIK3R2 |
| KEGG | Osteoclast differentiation | 2 | 0.0225 | PIK3R2; IL1A |
| KEGG | Measles | 2 | 0.0235 | PIK3R2; IL1A |
| KEGG | Insulin signaling pathway | 2 | 0.0255 | PIK3R2;SH2B2 |
| Wikipathways | BDNF signaling pathway | 2 | 0.0268 | PIK3R2;SH2B2 |
| KEGG | Non-alcoholic fatty liver disease (NAFLD) | 2 | 0.0293 | PIK3R2; IL1A |
| KEGG | Influenza A | 2 | 0.0384 | PIK3R2; IL1A |
