# Supplementary material for: Gene Co-Expression Network Analysis for Identifying Modules and Functionally Enriched Pathways in Type 1 Diabetes
Source: PLoS One. 2016 Jun 3;11(6):e0156006. doi: 10.1371/journal.pone.0156006 (PMC4892488; doi:10.1371/journal.pone.0156006)
Supplement: S8 Table — Table shows resulting KEGG pathways enriched in Pink module. (DOC) [file pone.0156006.s008.doc]

S8 Table. Pathway enrichment results for Pink module.

| Source | Pathway | Count | p-value | Genes |
| --- | --- | --- | --- | --- |
| KEGG | HTLV-I infection | 5 | 0.000566 | DLG1; NFATC3; XPO1; TGFBR1; CRTC3 |
| KEGG | T cell receptor signaling pathway | 2 | 0.0314 | NFATC3; DLG1 |
| KEGG | Chagas disease | 2 | 0.0452 | CFLAR; TGFBR1 |
